# Da Vinci robot-assisted retroperitoneal tumor resection in 105 patients: a single-center experience

**DOI:** 10.3389/fonc.2024.1414780

**Published:** 2024-07-23

**Authors:** Qisheng Hao, Lichao Cha, Bin Zhou, Xinyu Li, Mingkai Gong, Qingze Li, Guofei Dong, Mengqi Song, Zehua Wu, Zhongyi Guo, Fabo Qiu, Xiaowei Wang, Lantian Tian

**Affiliations:** ^1^ Department of Hepatobiliary and Pancreatic Surgery, The Affiliated Hospital of Qingdao University, Qingdao, Shandong, China; ^2^ Department of Retroperitoneal Tumor Surgery, Affiliated Hospital of Qingdao University, Qingdao, Shandong, China; ^3^ Department of Gastroenterology, The Affiliated Hospital of Qingdao University, Qingdao, Shandong, China

**Keywords:** retroperitoneal tumor, robotic surgery, minimally invasive technique, Da Vinci surgery system, robot-assisted retroperitoneal tumor resection

## Abstract

**Background:**

The Da Vinci Surgical System (DVSS) has the advantages of minimal invasion, rapid recovery, safety, and reliability. Although the DVSS has been widely used in various abdominal surgeries, descriptions of its use in robot-assisted retroperitoneal tumor resection (RRTR) are limited to case reports; large-sample systematic studies are lacking. The present study was performed to analyze the data of RRTR in our center, summarize our experience, and provide a reference for other retroperitoneal tumor centers.

**Methods:**

We retrospectively analyzed the clinical data of 105 patients who underwent RRTR at the Affiliated Hospital of Qingdao University from January 2015 to December 2022. Logistic univariate and multivariate analyses were performed to identify independent risk factors affecting RRTR. A receiver operating characteristic curve was used to find the cut-off value, which was then included in the logistic multivariate analysis for verification.

**Results:**

Among the 105 patients, 87 successfully underwent RRTR (DVSS group) and 18 underwent conversion to open surgery (conversion group). There was no significant difference in sex, age, body mass index, history of abdominal surgery, or tumor location between the two groups (P > 0.05). The maximum tumor diameter [odds ratio (OR), 1.041; 95% confidence interval (CI), 1.015-1.067; P = 0.002] and pathological property (OR, 8.646; 95% CI, 2.370-31.544; P = 0.001) were independent risk factors for conversion to open surgery. Further analysis confirmed that the success rate of RRTR was higher for tumors with a maximum diameter of ≤64 mm and benign tumors. Based on our experience and statistical results, we believe that retroperitoneal tumors that meet the following criteria have a higher success rate of DVSS resection: maximum tumor diameter of ≤64 mm, benign tumors, the tumor has relatively clear boundary, no obvious invasion of surrounding tissues and organs, and no need for combined organ resection.

**Conclusions:**

RRTR is safe and effective in the treatment of RPT, and the clinical prognosis is similar to that of open surgery. The success rate of RRTR in patients with appropriate surgical indications for this procedure is higher.

## 1 Introduction

A retroperitoneal tumor (RPT) arises from fat, muscle, lymph, nerve, and residual embryonic tissue. These tumors may be located anywhere within the retroperitoneal space, which extends from the plane of the diaphragm to the potential retroperitoneal space above the pelvis (1). Statistical data indicate that the incidence of RPT ranges from 0.5 to 1.0 per 100,000 individuals (2). Malignant retroperitoneal tumors constitute approximately 70% of all RPTs and account for 0.1%–0.2% of all human malignancies (3). Despite their rarity, about one-third of RPTs are sarcomas, which are associated with an extremely poor prognosis and high recurrence rates (4). Surgical intervention remains the primary treatment modality for RPT and is a crucial factor in determining patient outcomes (5, 6). The main surgical techniques for RPT include traditional open surgery, laparoscopic approaches, and robot-assisted procedures [including the use of the Da Vinci Surgical System (DVSS) (Intuitive Surgical, Sunnyvale, CA, USA)]. The challenges presented by the limited surgical space, narrow surgical field, restricted surgical range, deep tumor location, various pathological types, and proximity to blood vessels in the retroperitoneal space have been addressed by increasing numbers of surgeons specializing in retroperitoneal tumors. These surgeons have demonstrated the safety and effectiveness of open surgery for RPT resection through good exposure of the surgical field and the ability to identify tumors by intraoperative palpation. Therefore, traditional open surgery is widely performed for retroperitoneal tumor resection. However, traditional open surgery cannot avoid the need for a large surgical incision, intraoperative manipulation of organs, slow postoperative recovery, and potential complications; thus, clinicians still face many challenges in perioperative management. With the development of minimally invasive techniques in recent years, successful laparoscopic treatment of RPT has been reported, and many retroperitoneal tumor surgeons continue to progress in this field (7, 8). For example, Chatelet et al. (9) laparoscopically removed a large schwannoma measuring 17 × 8 × 6 cm. Laparoscopic RPT resection is feasible even when the tumor is large or attached to adjacent vascular structures, and although several challenges remain (10), laparoscopic surgery is technically safe, improves patients’ prognosis, and is a viable surgical option (11). The development of robot-assisted surgical systems is one of the greatest advances in laparoscopic technology. Several reports have confirmed that the DVSS is safe and effective for RPT resection and that it can significantly reduce surgical trauma and promote patient recovery (12–19). However, no systematic, large-sample studies have been performed to evaluate the application of DVSS robot-assisted RPT resection (RRTR). The present study was performed to analyze the clinical data of patients who underwent RRTR in our hospital, identify the risk factors affecting RRTR, and provide a reference for the application of RRTR in other retroperitoneal tumor centers.

## 2 Materials and methods

This study involved 105 patients who underwent RRTR at the Affiliated Hospital of Qingdao University from January 2015 to December 2022. Preoperative color Doppler ultrasound, contrast-enhanced computed tomography or magnetic resonance imaging, endoscopic ultrasonography, and three-dimensional imaging were used to comprehensively diagnose and evaluate the resectability of RPT. All patients were evaluated by a multidisciplinary treatment team before surgery, and the surgical plan was formulated. Preoperatively, patients were informed in detail of the surgical plan and the possibility of conversion to open surgery. This study was approved by the Institutional Review Board of the Affiliated Hospital of Qingdao University.

### 2.1 Patient selection

The inclusion criteria for the study were relatively clear tumor boundaries observed during preoperative examinations, indicating that complete resection was feasible based on preoperative assessments, absence of preoperative anesthetic or surgical contraindications, no evidence of metastasis, and no prior exposure to preoperative chemoradiotherapy or targeted therapy. The exclusion criteria were severe uncontrolled infection; tumor recurrence; unsuitability for surgery because of severe cardiovascular or cerebrovascular disease, hematological disease, immune system disease, or diabetes; and pregnancy or lactation.

### 2.2 Perioperative data

The basic data, perioperative information, and pathological reports of all patients who had successfully undergone RRTR, recovered, and been discharged were obtained from the electronic medical records. The operation time, estimated blood loss, blood transfusion volume, and postoperative complications were analyzed.

Data on the tumor location, number of tumors, pathological properties, maximum tumor diameter, and adhered to major blood vessels were obtained from imaging and pathology reports. Tumor adhered to major vessels was defined as tumor contact with the great vessels, including the aorta, inferior vena cava, portal vein, renal artery and vein, splenic artery and vein, and superior mesenteric artery and vein. Postoperative complications were graded according to the Clavien–Dindo classification.

### 2.3 Surgical technique and follow-up

All procedures were performed with the DVSS by the same team of surgeons who had received standardized training in robotic surgery and were certified to perform the procedure. The surgical position varied according to the patient’s body size, body mass index, and tumor location. The supine position or the contralateral 70° lateral decubitus position was chosen to establish pneumoperitoneum, insert a trocar, and install a robotic arm. After ensuring that no metastasis was present, the tumor was exposed, carefully separated along the tumor capsule, completely removed, and loaded into a specimen bag. The specimen was then removed for routine examination. All patients were followed up at the outpatient clinic 1 month after discharge and every 3 months thereafter.

### 2.4 Statistical analysis

SPSS 24 software (IBM Corp., Armonk, NY, USA) was used for statistical analysis. Continuous variables are expressed as mean ± standard deviation and were compared using the t test. Categorical variables are expressed as count ratio and were compared using the chi-square test. In total, 105 patients who underwent RRTR were divided into the DVSS group and the conversion group according to whether they had undergone conversion to open surgery. Correlations between parameters were analyzed. Logistic univariate and multivariate analyses were performed to identify independent risk factors affecting the need for conversion to open surgery. Logistic multivariate analysis was used to verify the results. A P value of <0.05 was considered statistically significant.

## 3 Results

### 3.1 Patient characteristics

Among the 105 patients, 87 successfully underwent RRTR (DVSS group) and 18 underwent conversion to open surgery (conversion group). The 105 patients comprised 40 men and 65 women. The baseline characteristics of the study population are shown in **Table 1**.

**Table 1:** Comparison of DVSS group and conversion group: demographic outcomes.

Parameter	DVSS group	Conversion group	P
	N=87	N=18	
Sex			0.253
Male, n (%) Female, n (%)	31 (35.6) 56 (64.4)	9 (50) 9 (50)	
Age, mean± SD	48.64 ± 10.52	51.33 ± 16.40	0.449
BMI, mean± SD	24.43 ± 2.86	24.10 ± 3.46	0.738
History of abdominal surgery, n (%)	20 (23.0)	3 (16.6)	0.555
Adhered to major vessels, n (%)	46 (52.9)	9 (50)	0.824
Tumor number, mean± SD	1.0 ± 0	1.0 ± 0	
Maximum tumor diameter (mm), mean± SD	46.45 ± 16.08	62.56 ± 23.42	0.006

BMI, body mass index; SD, standard deviation.

### 3.2 Pathological outcomes

**Figure 1:**
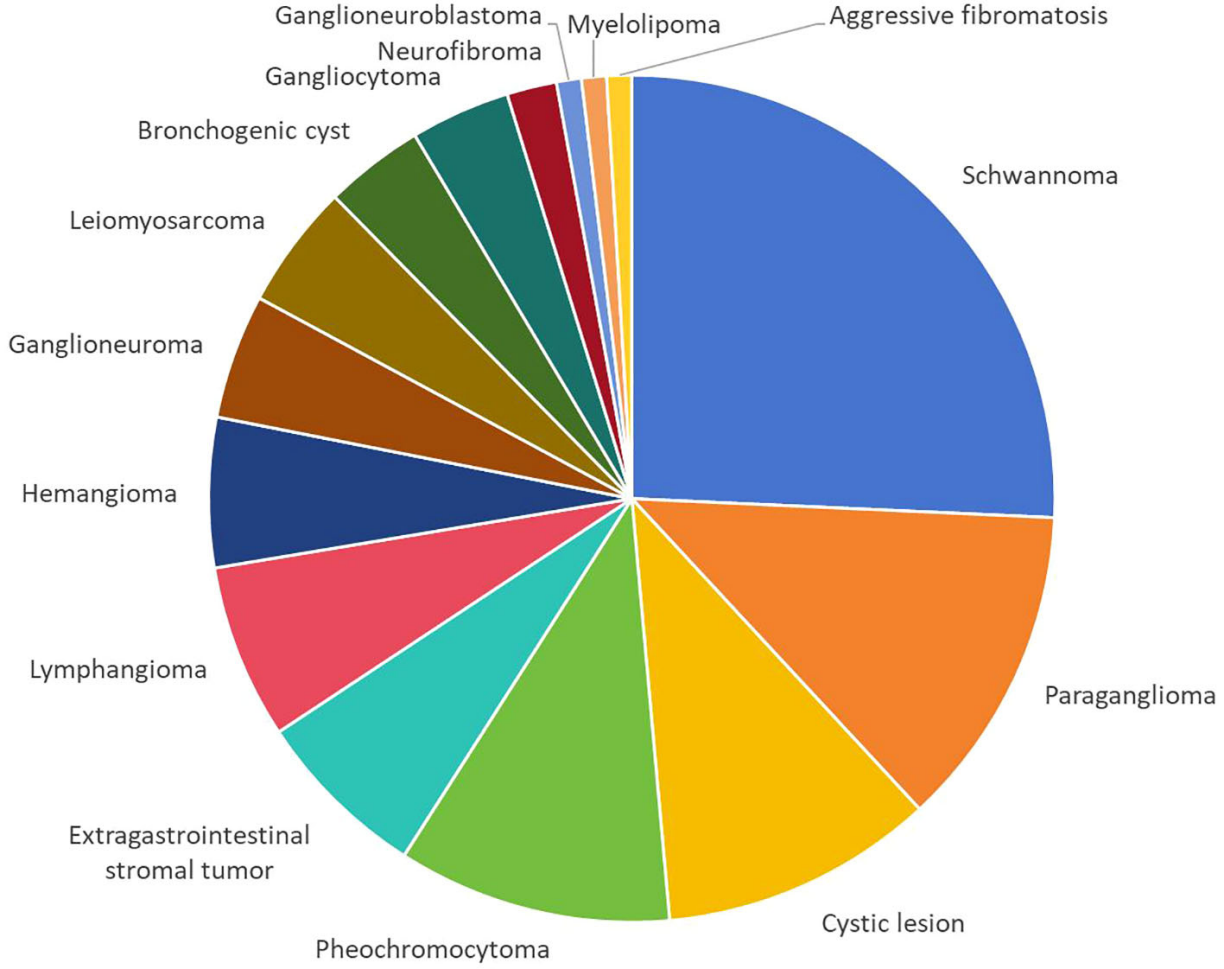
Pathological findings of the study population.

The pathological results were based on the final histopathology, which revealed 37 malignant tumors and 68 benign tumors. As shown in **Figure 1**, schwannoma (n = 27) was the most common, followed by paraganglioma (n = 13), cystic lesion (n = 11), pheochromocytoma (n = 11), extragastrointestinal stromal tumor (n = 7), lymphangioma (n = 7), hemangioma (n = 6), ganglioneuroma (n = 5), leiomyosarcoma (n = 5), bronchogenic cyst (n = 4), gangliocytoma (n = 4), neurofibroma (n = 2), ganglioneuroblastoma (n = 1), myelolipoma (n = 1), and aggressive fibromatosis (n = 1).

### 3.3 Comparison of DVSS group and conversion group: perioperative outcomes

The patients’ perioperative characteristics are shown in **Table 2**. No intraoperative complications occurred in the DVSS group. One patient received 4 U red blood cells during the operation, and one patient developed a postoperative complication (unilateral atelectasis). One patient in the conversion group had intraoperative blood loss of 2400 mL, received an intraoperative transfusion of 8 U red blood cells and 840 mL plasma, and received a postoperative transfusion of 570 mL plasma. One patient developed a postoperative complication (pancreatic fistula, which healed after nonsurgical treatment).

**Table 2:** Comparison of DVSS group and conversion group: perioperative outcomes.

Parameter	DVSS group	Conversion group	P
	N=96	N=18	
Operative time (minutes), mean± SD	163.50±62.91	212.22±88.39	0.006
Estimated blood loss (mL), mean± SD	30.28±84.27	230.00±556.35	0.001
Blood transfusion, n (%)	1 (1.0)	1 (5.6)	0.292
Pathological property			
malignant tumor, n (%)	27 (28.1)	12 (66.7)	0.002
ASA score, n (%)			0.373
1	74 (77.1)	14 (77.8)	
2	21 (21.9)	3 (16.7)	
3	1 (1.0)	1 (5.6)	
4	0 (0)	0 (0)	
Tumor location, n (%)			0.7121
Right upper area	26 (27.1)	5 (27.8)	
Left upper area	45 (46.9)	6 (33.3)	
Right lower area	9 (9.4)	3 (16.7)	
Left lower area	14 (14.6)	3 (16.7)	
Pelvic	2 (2.1)	1 (5.6)	
Intraoperative complications, n(%)	0 (0)	1 (5.6)	
Postoperative complications, n(%)			
Clavien I-II	1 (1.04)	0 (0)	
Clavien ≥III	0 (0)	1 (5.56)	
Postoperative hospital stay (days), mean± SD	3.36±2.03	6.11±6.16	0.001
Surgical margins, n (%)			
Positive margin	0 (0)	0 (0)	
Negative margin	96 (100)	18 (100)	
Hospitalization expenses, mean± SD	59940.43±9243.68	68230.22±20168.35	0.006
90-day readmission	0 (0)	0 (0)	
90-day mortality	0 (0)	0 (0)	
Reoperation	0 (0)	0 (0)	

ASA, American Society of Anesthesiologists; SD, standard deviation.

There were no significant differences in sex (P = 0.253), age (P = 0.449), body mass index (P = 0.738), history of abdominal surgery (P = 0.555), or tumor adhesion to large vessels (P = 0.824) between the DVSS group and conversion group. However, there were significant differences in the maximum tumor diameter (P = 0.006), pathological property (P = 0.002), blood loss (P = 0.002), operation time (P = 0.037), and postoperative hospital stay (P = 0.026). There was no significant difference in the frequency of blood transfusion between the two groups (1.1% vs. 5.6%, P = 0.281). The operation time in the DVSS group was significantly shorter than that in the conversion group (163.28 ± 47.76 vs. 212.22 ± 88.39 min, P = 0.037). Additionally, the blood loss volume was lower (31.69 ± 32.56 vs. 230.00 ± 556.35 mL, P = 0.002) and the postoperative hospital stay was shorter (3.62 ± 1.11 vs. 6.11 ± 6.16 days, P = 0.026) in the DVSS group. The hospitalization cost was higher in the conversion group (60441.33 ± 7047.89 vs. 68230.22 ± 10168.35 yuan, P = 0.046).

No patient required reoperation or readmission, and the 90-day mortality rate was 0%. The median follow-up time was 19 months (range, 13–36 months) and there was no imaging evidence of tumor recurrence in any patient.

### 3.4 Regression analysis

**Figure 2:**
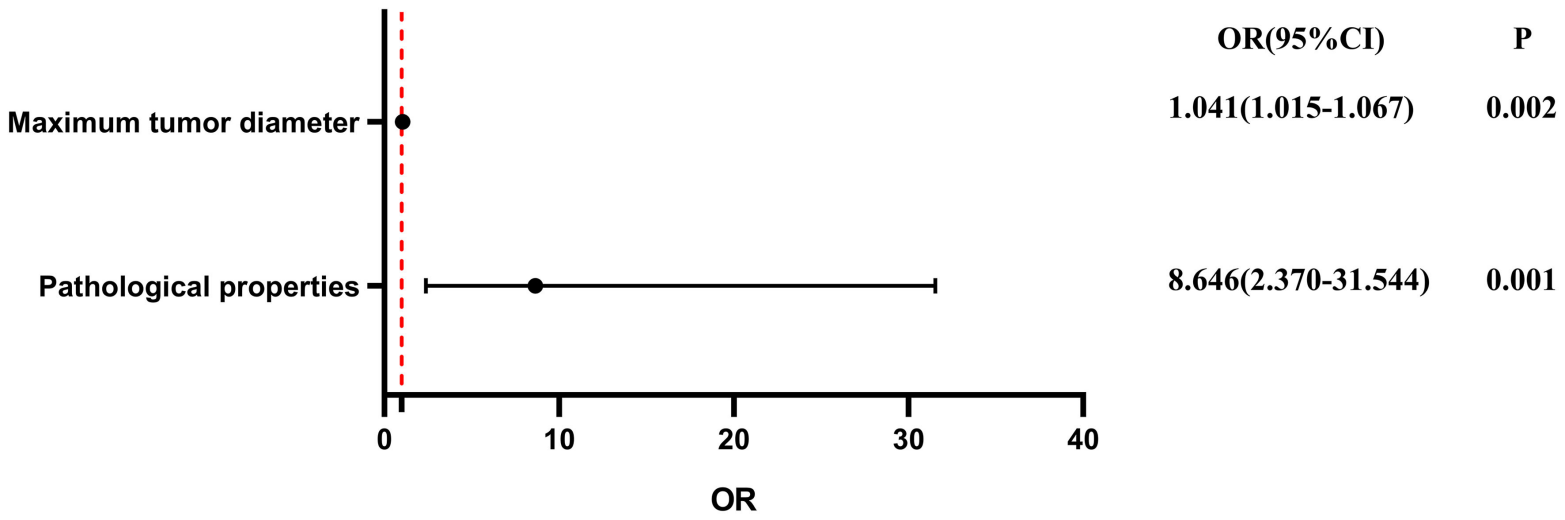
Multivariate logistic regression analysis of conversion from Da Vinci surgery to open surgery. OR, odds ratio; CI, confidence interval.

**Figure 3:**
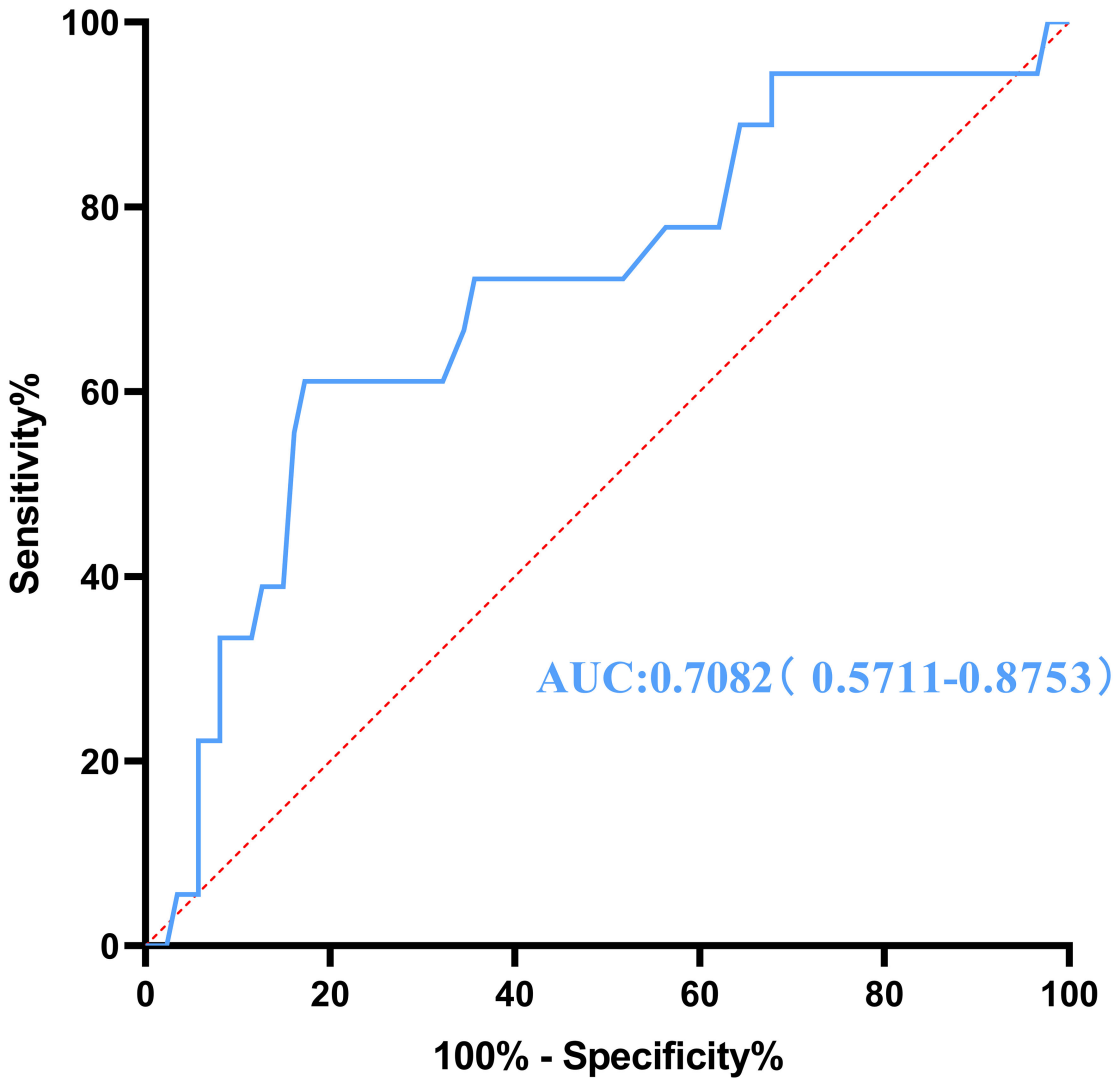
Receiver operating characteristic curve for conversion to open surgery generated according to the maximum tumor diameter. AUC, area under the curve.

**Figure 4:**
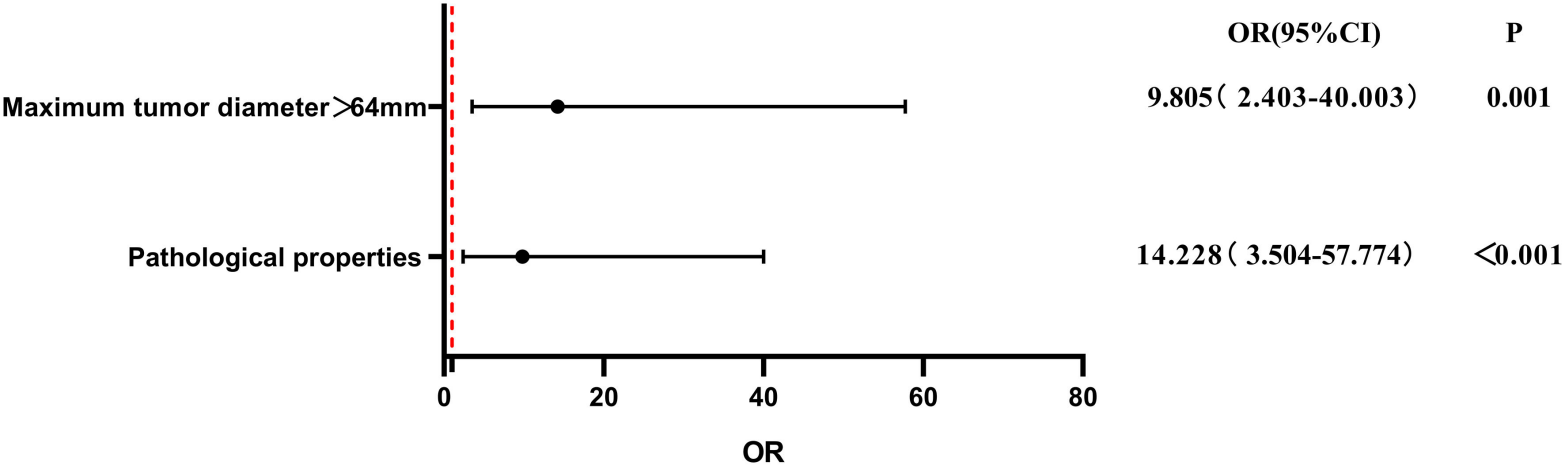
Multivariate logistic regression analysis of risk factors for conversion to open surgery after classification by cut-off value. OR, odds ratio; CI, confidence interval.

To evaluate the influence of various factors on the need for conversion to open surgery, relevant parameters were included in the univariate logistic regression analysis to screen out risk factors and further included in the multivariate logistic regression analysis. The results showed that the independent risk factors for conversion to open surgery were the pathological property of the tumor [odds ratio (OR), 8.646; 95% confidence interval (CI), 2.370-31.544; P = 0.001] and maximum tumor diameter (OR, 1.041; 95% CI, 1.015-1.067; P = 0.002) (**Figure 2**, **Table 3**). These findings indicate that a larger maximum tumor diameter is associated with a higher probability of conversion to open surgery and that the probability of conversion to open surgery is higher for malignant than benign tumors. **Figure 3** shows the receiver operating characteristic curve generated according to the maximum tumor diameter, with a determined cut-off value of 64 mm for conversion to open surgery. The classification variable and pathological property were used as covariates, and the decision to convert to open surgery served as the dependent variable for logistic regression analysis. The Hosmer test indicated a good fit for the model (P = 0.787 > 0.05). The pathological property of the tumor (OR, 9.805; 95% CI, 2.403-40.003; P = 0.001) and a maximum tumor diameter of >64 mm (OR, 14.228; 95% CI, 3.504–57.774; P < 0.001) were independent risk factors for conversion to open surgery (**Table 4**, **Figure 4**). These findings indicate that RRTR has a higher success rate for retroperitoneal benign tumors with a maximum diameter of ≤64 mm.

**Table 3:** Univariate and multivariate logistic regression analysis of conversion from Da Vinci robotic surgery to open surgery.

Parameter	Univariate analysis		Multivariate analysis	
	OR (95% CI)	P	OR (95% CI)	P
Sex	2.537 (0.740-8.704)	0.139		
Age	1.017 (0.970-1.065)	0.490		
BMI	0.903 (0.760-1.072)	0.242		
History of abdominal surgery	1.455 (0.334-6.341)	0.617		
Maximum tumor diameter	1.047 (1.018-1.077)	0.001	1.041(1.015-1.067)	0.002
Adhered to major vessels	1.068 (0.302-3.774)	0.919		
Pathological property	8.382 (2.220-32.882)	0.002	8.646(2.370-31.544)	0.001

BMI, body mass index; OR, odds ratio; CI, confidence interval.

**Table 4:** Multivariate logistic regression analysis of risk factors for conversion to open surgery after classification by cutoff value.

Parameter	OR	95% CI		P
		Lower limit	Upper limit	
Pathological property	9.805	2.403	40.003	0.001
Maximum tumor diameter > 64mm	14.228	3.504	57.774	<0.001

OR, odds ratio; CI, confidence interval.

### 3.5 Tumor location and intraoperative status of the conversion group

In this study, the total conversion rate was 17.14%. As shown in **Table 5**, the reasons for conversion included poor vision (7 cases), uncontrolled bleeding (4 cases), difficulties in achieving radical resection with DVSS (5 cases), and the occurrence of hypertensive crisis during surgery (2 cases). In the conversion group, there were 8 cases of combined organ resection. Given the proximity to the inferior vena cava (IVC), 2 cases of right upper abdominal RPT were challenging to separate, leading to partial resection and repair of the IVC during surgery. One case of right upper abdominal RPT required partial duodenectomy because of its extensive invasion into the duodenum. Another case of left upper abdominal RPT, situated between the left kidney and pancreas and closely associated with the tail of the pancreatic body and left kidney, necessitated distal pancreatectomy. Additionally, 1 case of left upper abdominal RPT involved the gastric antrum and was managed with partial gastrectomy. One case of RPT in the right lower abdominal mesenteric region required partial small intestine resection and enterostomy. The left lower abdominal RPT in 2 cases was challenging to separate from the left psoas major muscle, resulting in partial resection of this muscle during surgery.

**Table 5:** Tumor location and intraoperative status of conversion group.

Tumor location	Specific location	Maximum tumor diameter (mm)	Pathological outcome	Pathological property	Conversion reason	Combined organ resection
**Right upper area**	Behind the IVC	23	Pheochromocytoma	Malignant tumor	Hypertensive crisis occurred during the operation	Partial resection and repair of the IVC
	Below the horizontal part of the duodenum	36	Extragastrointestinal stromal tumor	Malignant tumor	Radical resection is difficult with DVSS	Partial duodenectomy
	Below the right renal hilum	70	Pheochromocytoma	Malignant tumor	Uncontrolled bleeding	
	Behind the IVC	34	Paraganglioma	Malignant tumor	Poor visualization	
	Right side of the IVC	66	Leiomyosarcoma	Malignant tumor	Radical resection is difficult with DVSS	Partial resection and repair of the IVC
**Left upper area**	Below the left renal hilum	40	Pheochromocytoma	Malignant tumor	Poor visualization	
	Below the bifurcation of the abdominal aorta	66	Pheochromocytoma	Malignant tumor	Uncontrolled bleeding	
	Between the left kidney and the pancreas	66	Leiomyosarcoma	Malignant tumor	Radical resection is difficult with DVSS	Distal pancreatectomy
	Left side of gastric antrum	36	Extragastrointestinal stromal tumor	Malignant tumor	Radical resection is difficult with DVSS	Partial gastrectomy
	Left side of the IVC	50	Pheochromocytoma	Malignant tumor	Hypertensive crisis occurred during the operation	
	Behind the tail of the pancreas	74	Hemangioma	Benign tumor	Uncontrolled bleeding	
**Right lower area**	Mesenteric area	89	Aggressive fibromatosis	Malignant tumor	Poor visualization	Small intestinal resection-anastomosis
	Behind of ascending colon	87	Hemangioma	Benign tumor	Uncontrolled bleeding	
	Mesenteric area	90	Lymphangioma	Benign tumor	Poor visualization	
**Left lower area**	Right side of the psoas major muscle	75	Cystic lesion	Benign tumor	Poor visualization	
	In front of the psoas major muscle	65	Leiomyosarcoma	Malignant tumor	Radical resection is difficult with DVSS	Partial resection of the psoas muscle
	In front of the psoas major muscle	110	Neurilemmoma	Benign tumor	Poor visualization	Partial resection of the psoas muscle
**Pelvic**	In front of the sacrum	49	Neurilemmoma	Benign tumor	Poor visualization	

## 4 Discussion

The DVSS is currently the most widely used robotic system. It has the advantages of magnified and stable three-dimensional stereovision, multidimensional robotic arm movement, elimination of hand tremor, flexible instruments to enhance tactile feedback, no need for reverse operation or intensive training of operators and assistants, and the potential for use in remote surgery. It has the same surgical efficacy as laparoscopic techniques in the treatment of various diseases, and it overcomes several limitations of laparoscopic surgery such as the lack of three-dimensional depth perception in two-dimensional imaging, high technical difficulty, and a steep learning curve (20–23). Widespread use of the DVSS can also reduce reliance on surgical assistants, thereby reducing the number of assistants and saving medical resources (24). At the same time, competition between hospitals and increased patient expectations have contributed to the popularity of robot-assisted surgery (25). Surgical resection of RPT is often accompanied by a variety of postoperative complications due to the limited working space and rich, fine structures within the retroperitoneal space, which prompts retroperitoneal tumor surgeons to attempt resection with minimal trauma and the shortest possible operation time. It is quite helpful to use robotic techniques that are finer, safer, and more stable than traditional laparoscopic surgery. Such techniques can significantly reduce damage to the surrounding tissues and the occurrence of complications (19, 26). However, no systematic large-sample case study on RRTR has been performed to date. To further explore the feasibility of RRTR and promote safer use of robotic technology, we analyzed the data of 105 patients who underwent RRTR in our hospital and identified risk factors for conversion to open surgery. With this study, we aim to provide a preliminary basis for preoperative evaluation of surgical indications and guide clinical practice. To the best of our knowledge, this is the first large-sample study of RRTR.

In total, 105 patients who underwent RRTR were included in this study. Among them, 87 patients successfully underwent RRTR (DVSS group) and 18 patients underwent conversion to open surgery (conversion group). There were no significant differences in sex (P = 0.253), age (P = 0.449), body mass index (P = 0.738), history of abdominal surgery (P = 0.555), or tumor adhered to major vessels (P = 0.824) between the DVSS group and conversion group, although patients with a history of abdominal surgery inevitably had different degrees of intestinal adhesion. Changes in the normal anatomical structure of the abdomen and tumor adhesion to large abdominal blood vessels will also increase the difficulty of the operation, but these are not key reasons for conversion of robotic to open surgery. Using the DVSS, the main blood vessels around the tumor can be accurately dissected and bleeding can be controlled. This approach is beneficial in terms of the tumor anatomy and may be more suitable for RRTR. This is consistent with the results reported by Liu (12). In our study, the procedure time was shorter in the DVSS than conversion group (163.28 ± 47.76 vs. 212.22 ± 88.39 min, P = 0.037). Additionally, the blood loss volume was lower (31.69 ± 84.27 vs. 230.00 ± 556.35 mL, P = 0.002) and the postoperative hospital stay was shorter (3.62 ± 1.11 vs. 6.11 ± 6.16 days, P = 0.026) in the DVSS group. The dexterity and precision of the DVSS can reduce surgical trauma (27, 28), and minimally invasive surgery can reduce postoperative pain (29, 30); both of these factors promote faster patient recovery after RRTR. Conversion to open surgery requires a change of surgical instruments, which may have contributed to the increased operation time in the conversion group; however, this longer operation time may not be clinically significant.

This study revealed that the pathological property of the tumor (OR, 9.805; 95% CI, 2.403-40.003; P = 0.001) and maximum tumor diameter of >64 mm (OR, 14.228; 95% CI, 3.504–57.774; P < 0.001) were independent risk factors for conversion to open surgery. Higher success rates are observed in benign retroperitoneal tumors with a maximum diameter of ≤64 mm. Therefore, the pathological property and size of the tumor should be determined according to preoperative imaging examination or biopsy, which is helpful for evaluating the difficulty of the operation and provides a preliminary basis for clinical and surgical decision-making. A study by the Transatlantic Australasian Retroperitoneal Sarcoma Working Group showed that schwannomas increase in size at a rate of 10.5% per year (31). If the tumor size increases by ≥20% per year, surgical resection is recommended regardless of the presence or absence of symptoms, and the success rate of R0/R1 resection for benign RPT is as high as 91.6% (32). Therefore, patients with asymptomatic retroperitoneal tumors detected by physical examination should be actively treated with surgery because of the unpredictability of tumor growth and the possible progression to malignancy. The present study indicates that minimally invasive surgery is preferable for benign tumors measuring ≤64 mm in diameter, with RRTR being the treatment of choice for these lesions.

In the present study, the total conversion rate was 17.14%, with the rate of conversion to open surgery for malignant tumors standing at 32.43%. Moreover, two-thirds of the tumors treated by conversion to open surgery were malignant. The reasons for conversion to open surgery, consistent with findings from other studies (33), included poor visibility (seven cases), uncontrolled bleeding (four cases), difficulty achieving radical resection with DVSS (five cases), and the occurrence of hypertensive crisis during surgery (two cases). In instances where malignant tumors could not be radically cured through RRTR, our team promptly performed conversion to open surgery. Retroperitoneal malignant tumors, noted for their invasiveness, necessitate a wider resection margin to ensure negative margins, thereby increasing the risk of damage to surrounding tissues and the possibility of incomplete resection. Given these considerations and the implications for patient prognosis, open surgery remains the recommended approach because of its safety (34). The need for combined organ resection often arises under several circumstances: 1. suspected tumor invasion; 2. tumor involvement in the peripheral vascular supply of organs; 3. tumors encasing or adhering to organs, making separation difficult; and 4. iatrogenic injuries necessitating organ resection. In our study, the Da Vinci group, consisting of 96 cases, did not report any intraoperative combined organ resections. However, in the conversion group, which included 18 cases, 8 required intraoperative combined organ resections. Of these, five involved tumors in the upper abdomen, where vital organs such as the liver, kidneys, pancreas, spleen, and duodenum are located alongside major vessels like the inferior vena cava (IVC), abdominal aorta, hepatic hilum, and renal hilum, presenting complex anatomical challenges and surgical difficulties. There were three cases of combined organ resection for tumors in the lower abdomen, which includes structures such as the colon, small intestine, mesentery, psoas major, and related vessels. During surgical dissection in these cases, it is crucial to protect the ureter, mesenteric vessels, and iliac vessels. Additionally, in cases where pheochromocytoma is suspected, preparations to convert to open surgery should be made intraoperatively, as approximately 15% of patients may experience hemodynamic instability or crisis despite adequate preoperative preparation (35).

We recommend conversion to open surgery when complications occur during RRTR, the clinician suspects incomplete tumor resection, or the intraoperative pathology suggests malignancy requiring wide resection. Especially for malignant tumors, the following principles of radical tumor treatment must be followed: complete resection of the tumor and surrounding tissue, minimization of direct physical manipulation of the tumor (non-contact principle of tumor surgery), achievement of adequate margins, and complete lymph node dissection (36).

Retroperitoneal tumors with a diameter of >64 mm will have a limited surgical field, narrow surgical space, and increased difficulty of surgery; thus, they are more suitable for open surgery. However, tumor size is not an absolute contraindication for minimally invasive surgery (37). For example, a retroperitoneal tumor with a maximum diameter of 131 mm was removed by RRTR in our center. Thus, even if the tumor is large, RRTR can still be considered based on factors such as whether the tumor is easy to expose. Notably, for huge or malignant tumors, the increased operation time may introduce additional risks such as anesthetic complications, pulmonary infections, and postoperative nursing challenges. From doctors’ perspective, striving for high rates of minimally invasive procedures is valuable but should not be done at the expense of patient safety.

Although the learning curve of RRTR is unknown, it should not be ignored. Mastering RRTR is indeed a challenging undertaking, and surgeons are advised to proceed with great caution even if they are already very familiar with open and laparoscopic RPT procedures.

The DVSS has the disadvantages of a long training time, long setup time, long operation time, and high cost, all of which limit its application. The high costs associated with using the DVSS are mainly related to the purchase and maintenance of the equipment, the high cost of the instruments, and the long operating time. Although the main limitation of using the DVSS is the additional cost to the patient, this may be offset by the benefits of reduced trauma and bleeding, a shorter hospital stay, and an earlier return to work. With the emergence of increasingly more new robotic systems, such as the avatera^®^ robotic system (avateramedical GmbH, Jena, Germany) and the hinotori™ robotic system (Medicaroid, Kobe, Japan), the cost and limitations of robotic surgery will gradually decrease (38, 39). Its wide applicability is likely to facilitate further substantial progress over the next decade. Reasonable selection of surgical methods can improve resource utilization and reduce costs for patients and the medical system. As a complex surgical method, RRTR must be explored in detail to help clinicians make informed decisions and benefit more patients.

This was a retrospective study and has certain inherent limitations. First, this study involved a single-center retrospective analysis. To the best of our knowledge, this is the first large-sample study of RRTR; nevertheless, the number of cases was limited, increasing the risk of statistical bias. Multicenter prospective studies are needed to confirm the conclusions drawn in this study. Second, retroperitoneal tumors are clinically rare, and the sample size of this study is low; this may reduce the reliability of the final results to some extent. Third, there may have been errors in the data obtained from the medical records, such as the anesthetic details, operation time, and blood loss, and such errors may have affected the statistical results. Fourth, younger patients with a higher socioeconomic status or better health status may be more inclined to choose robot-assisted surgery, which may lead to selection bias. Fifth, patients who received neoadjuvant therapy before surgery were not included in this study; therefore, whether neoadjuvant chemotherapy or radiotherapy affects the DVSS procedure remains unclear.

## 5 Conclusion

RRTR is safe in experienced centers, and its clinical prognosis is similar to that of open surgery. Patients with retroperitoneal tumors who undergo RRTR have a higher chance of surgical success when the maximum tumor diameter is ≤64 mm, the tumor is benign, the tumor has relatively clear boundary, there is no obvious invasion of surrounding tissues and organs, and there is no need for combined organ resection.

## Data availability statement

The original contributions presented in the study are included in the article/supplementary material. Further inquiries can be directed to the corresponding authors.

## Ethics statement

The patients involved in the database have obtained Medical Ethics Committee of The Affiliated Hospital of Qingdao University approval (QYFY WZLL 28271). The studies were conducted in accordance with the local legislation and institutional requirements. The participants provided their written informed consent to participate in this study. Written informed consent was obtained from the individual(s), and minor(s)’ legal guardian/next of kin, for the publication of any potentially identifiable images or data included in this article.

## Author contributions

QH: Data curation, Formal analysis, Software, Writing – original draft. LC: Writing – original draft, Writing – review & editing. BZ: Methodology, Resources, Writing – review & editing. XL: Data curation, Writing – original draft. MG: Data curation, Writing – original draft. QL: Data curation, Investigation, Writing – original draft. GD: Validation, Writing – original draft. MS: Investigation, Project administration, Writing – review & editing. ZW: Methodology, Project administration, Validation, Writing – review & editing. ZG: Data curation, Formal analysis, Writing – review & editing. FQ: Funding acquisition, Resources, Supervision, Writing – review & editing. XW: Methodology, Supervision, Writing – review & editing. LT: Funding acquisition, Resources, Writing – review & editing.
